# DyCeModel: a tool for 1D simulation for distribution of plant hormones controlling tissue patterning

**DOI:** 10.18699/VJGB-23-103

**Published:** 2023-12

**Authors:** D.S. Azarova, N.A. Omelyanchuk, V.V. Mironova, E.V. Zemlyanskaya, V.V. Lavrekha

**Affiliations:** Institute of Cytology and Genetics of the Siberian Branch of the Russian Academy of Sciences, Novosibirsk, Russia; Institute of Cytology and Genetics of the Siberian Branch of the Russian Academy of Sciences, Novosibirsk, Russia; Radboud Institute for Biological and Environmental Sciences (RIBES), Radboud University, Nijmegen, the Netherlands; Institute of Cytology and Genetics of the Siberian Branch of the Russian Academy of Sciences, Novosibirsk, Russia Novosibirsk State University, Novosibirsk, Russia; Institute of Cytology and Genetics of the Siberian Branch of the Russian Academy of Sciences, Novosibirsk, Russia Novosibirsk State University, Novosibirsk, Russia

**Keywords:** computer modeling, developmental trajectory, input data, genetic algorithm, phytohormones, компьютерное моделирование, траектория развития, входные данные, генетический алгоритм, фитогормоны

## Abstract

To study the mechanisms of growth and development, it is necessary to analyze the dynamics of the tissue patterning regulators in time and space and to take into account their effect on the cellular dynamics within a tissue. Plant hormones are the main regulators of the cell dynamics in plant tissues; they form gradients and maxima and control molecular processes in a concentration-dependent manner. Here, we present DyCeModel, a software tool implemented in MATLAB for one-dimensional simulation of tissue with a dynamic cellular ensemble, where changes in hormone (or other active substance) concentration in the cells are described by ordinary differential equations (ODEs). We applied DyCeModel to simulate cell dynamics in plant meristems with different cellular structures and demonstrated that DyCeModel helps to identify the relationships between hormone concentration and cellular behaviors. The tool visualizes the simulation progress and presents a video obtained during the calculation. Importantly, the tool is capable of automatically adjusting the parameters by fitting the distribution of the substance concentrations predicted in the model to experimental data taken from the microscopic images. Noteworthy, DyCeModel makes it possible to build models for distinct types of plant meristems with the same ODEs, recruiting specific input characteristics for each meristem. We demonstrate the tool’s efficiency by simulation of the effect of auxin and cytokinin distributions on tissue patterning in two types of Arabidopsis thaliana stem cell niches: the root and shoot apical meristems. The resulting models represent a promising framework for further study of the role of hormone-controlled gene regulatory networks in cell dynamics.

## Abstract (ru)

Для изучения механизмов роста и развития необходимо анализировать динамику распределения регуляторов по ткани во времени и пространстве и учитывать их влияние на клеточную динамику внутри ткани. Растительные гормоны являются основными регуляторами динамики клеток в тканях растений; они образуют градиенты и максимумы и контролируют молекулярные процессы в зависимости от концентрации. Мы представляем DyCeModel, программный инструмент, реализованный в среде MATLAB для одномерного моде лирования ткани с динамическим клеточным ансамблем, где изменения концентрации гормона (или другого активного вещества) в клетках описываются обыкновенными дифференциальными уравнениями. Мы применили DyCeModel для моделирования динамики клеток в меристемах растений с различной клеточной структурой и продемонстрировали, что DyCeModel помогает выявить взаимосвязь между концентрацией гормонов и поведением клеток. Инструмент визуализирует ход моделирования и предоставляет видео, полученное в ходе расчета. Важно отметить, что инструмент способен автоматически подбирать параметры, подгоняя распределение концентраций веществ, предсказанное в модели, к экспериментальным данным, полученным по изображениям с микроскопа. Примечательно, что DyCeModel позволяет строить модели для различных типов меристем растений на основе одних и тех же обыкновенных дифференциальных уравнений, используя для каждой меристемы специфические входные характеристики. Эффективность инструмента продемонстрирована путем моделирования влияния распределения ауксина и цитокинина на формирование паттерна ткани в двух типах ниш стволовых клеток Arabidopsis thaliana: апикальных меристемах корня и побега. Полученные модели представляют собой перспективный фреймворк для дальнейшего изучения роли контролируемых гормонами генных регуляторных сетей в динамике клеток

## Introduction

Understanding the control of cell division and differentiation in stem cell niches is among the major issues in plant developmental biology (Hayashi et al., 2023). Although many components of the molecular regulatory networks, which underlie these processes, have been identified, complex interactions and numerous players hinder detailed study on the mechanisms of their functioning. For example, it is still largely unknown how the formation of plant hormone concentration gradients results in particular alterations in the cellular dynamics of developing tissues and organs (Rutten et al., 2022). Dissection of these issues requires application of computer modeling to predict the output in cellular dynamics and to determine whether various developmental pathways exist under certain conditions (Fisher et al., 2023).

Nowadays, developmental biology has recruited experts in mathematical modeling and computer sciences to create appropriate tools. Numerical simulations were successfully used to study the influence of phytohormone concentration distribution on the functioning of plant stem cell niches in 1D and 2D models describing cell divisions, growth, and differentiation under control of signaling molecules (Kitano et al., 2005; Nikolaev et al., 2006; Mironova et al., 2010; Muraro et al., 2013; Band et al., 2014; De Rybel et al., 2014; Lavrekha et al., 2014; Dubreuil et al., 2018; Savina et al., 2020; Hartmann et al., 2021). At the same time, these models stay within the limits of a certain meristem, and are not applicable to a wider range of plant stem cell niches. A general description of the basic set of processes related to the redistribution of hormone gradients and cellular response to this may serve as a basis for the investigation of the common and specific features of various plant meristems.

To solve this kind of problem, professional tools have started to be developed, helping researchers to create extensible computer models, which enable applying the same mathematical model equations to various plant systems (Hay Mele et al., 2015; Schölzel et al., 2021). For example, Cell Designer is a tool for simulating biochemical networks (Kitano et al., 2005) without reference to the tissue topology. A similar tool, PySB, has ample opportunity to create, extend and combine models based on genetic networks with high complexity (Lopez et al., 2013). This Python-based software is highly flexible because it provides the possibility of direct manipulation of equations. BioNetGen allows to create models both using a graphic editor and describing models manually inside the program code that simplifies reconstruction of molecular networks (Harris et al., 2016). BioNetGen has a convenient graphical representation for the solution of equations. SBMLToolbox provides the possibility to create, validate and calculate models with ODEs using SBML in MATLAB and Octave (Keating et al., 2006). DBSolve features abundances of certain molecules in a system, displaying it dynamically as a bar graph (Gizzatkulov et al., 2010). MGSmodeller is a Java application, which enables hierarchical data presentation and editing, and implements dynamic calculation tools in reconstructing molecular genetic networks and solving inverse problems (Kazantsev et al., 2008). The COPASI software is able to describe models of biological processes, such as metabolic networks, cellular signaling pathways, regulatory networks, infectious diseases and many others, simulate and ana-lyze these models, create analysis reports and import/export models (reviewed in Bergmann et al., 2017). In COPASI, models are defined as chemical reactions between molecules. The model analyzer includes steady-state analysis, stoichiometric analysis, time history modeling using deterministic and stochastic modeling algorithms, metabolic control analysis, optimization and parameter estimation. VCell is a computing system for modeling physicochemical and electrophysiological processes in living cells (Loew, Schaff, 2001; Moraru et al., 2008). The tool allows the user to enter a description of cell physiology, biochemical reactions, and automatically or manually input mathematical equations. The resulting simulations are displayed on dynamic spatial regions of various shapes, including irregular 3D geometries derived from experimental images. VCell can also implement rule-based models, which allows the representation of species as structured objects consisting of molecules and uses reaction rules to define molecular interactions. SpringSaLaD is a software platform based on spatial stochastic modeling of biochemical systems (Michalski, Loew, 2016). SpringSaLaD models molecules as a group of connected spherical regions with excluded volume. This allows establishing a connection between molecular dynamics modeling and processes at the cellular level. SpringSaLaD is a standalone tool that supports model building, simulation, visualization, and data analysis through a graphical user interface

The tools listed above develop models for metabolic and signal transduction pathways, and gene regulation networks. Such tools do not implement embedding of the generated mathematical models into cell ensembles to study the influence of regulatory networks on cell divisions, growth and differentiation (Kitano et al., 2005; Keating et al., 2006; Kazantsev et al., 2008; Gizzatkulov et al., 2010; Lopez et al., 2013; Harris et al., 2016).

On the other hand, there are programs that along with simulation of gene networks also consider the influence of regulatory circuits on cell growth or divisions. CompuCell3D is a tool for constructing dynamic multicellular 2D and 3D models to simulate cells that lack a cell wall (Swat et al., 2012). It is based on the lattice-based Glazier–Graner–Hogeweg (GGH) Monte Carlo multi-cell modeling, which employs an energetic approach to model growth, intercellular communication and maintenance of cell shape. Molecular processes, namely, the production and diffusion of substances, are described via ODE solvers. The VirtualLeaf program simulates the relationship between gene expression and the biophysics of plant cell growth (Merks et al., 2011). The model is a set of cells and cell walls, through which chemical substances can move, affecting gene expression and properties of the cell wall. Cellzilla is a 2D tissue modeling platform using Cellerator, a tool describing biochemical interactions via simplified notation as reactions and converting them automatically to the corresponding differential equations by an inner computer algebra system (Shapiro et al., 2013). In Cellzilla, cells are represented by a polygonal grid of well-mixed compartments. Cell components can interact through Cellerator reactions, which describe diffusion and transport. Dynamic simulation consists of cell growth and division. Despite these advantages, modern software tools for modeling usually use manual setting of parameters, and do not support automatic parameter fitting, which may be critical for some models.

A recent trend is further improvement of computer tools, which can be used by biologists for in-depth study of developmental processes at the multicellular level. One of the current challenges is the creation of software that constructs numerical models along various plant organs utilizing uniformly described processes and provides automatic parameters setting. Here we present a tool creating one-dimensional computer models that provide embedding of signaling molecules into a dynamically developing cellular ensemble, where, based on the same set of processes, it is possible to model cellular dynamics in various plant tissues. To build realistic computer models, it is necessary to apply experimental data. The tool we have developed takes experimental data into account already at the first stage of parameter fitting, which brings the constructed models as close to reality as possible.

## Materials and methods

**Fig. 1.**
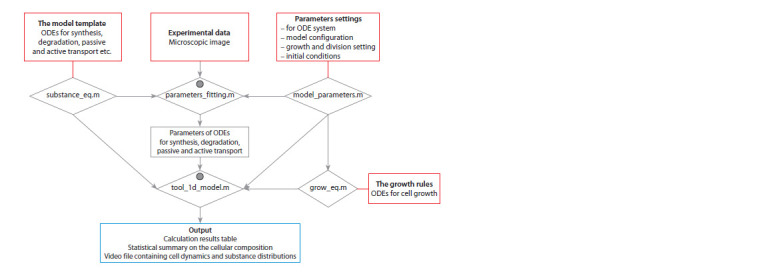
DyCeModel pipeline for creating mathematical models. The input data are marked in red. The output data are depicted in blue. Gray circles indicate the presence of visualization modules. Five script files are given in rhombuses.

DyCeModel overview. DyCeModel allows creating a dynamic one-dimensional cell lattice, embedding it into a mathematical model in ODE, and performing numerical analysis. It contains five script files (.m files) executed in the MATLAB software environment (Fig. 1). The substance_eq.m block incorporates an ODE system for description of synthesis, degradation, passive and active transport for the substances of interest. By default, DyCeModel provides examples of functions, which describe these processes for two substances according to Michaelis–Menten kinetics and Generalized Hill function method (Likhoshvai, Ratushny, 2007), Fick’s law of diffusion and the mass action law (for describing active transport). Alternatively, users can build their own functions instead of the default ones. The parameters_fitting.m block describes the realization of a genetic algorithm to assess the similarity of the modeled substance distribution to the experimental data. The model_parameters.m block contains all model parameter default values for the ODE system and describes the model configuration of substance influxes. The grow_eq.m block describes the cell growth function, tool_1d_model.m ensures the simulation procedure. Importantly, there are two different strategies of applying DyCeModel: using the parameters_fitting.m block or omitting it. In the latter case, the user should define all parameters in the model_parameters.m file.

The input data. A pre-processed experimentally obtained microscopic image, which visualizes the distribution profile of the substance concentration within the modeled tissue, is an input for the parameters_fitting.m block. DyCeModel accepts TIFF, GIF, JPEG, PNG formats and some other graphic file formats supported by MATLAB, and it is capable of processing the signal localized in the cytosol or in the nucleus. The image must be well focused. The aforementioned image preprocessing consists in excision of a rectangular area containing the modeled axis along the tissue, which should be parallel to the long side of the rectangle. This area should not contain microscope artifacts. To obtain noise-free measurements, the user can decrease the size of the rectangular area (the minimum size of the uploaded rectangular image is 1 pixel in width and 90 pixels in length). There are no strict requirements for image resolution.

The ODE system of the mathematical model is an input, which is written in the substance_eq.m file block (see Fig. 1). The default example equations can be changed according to the user’s request. The model configuration is defined by the initial number of cells and position of the substance influxes in model_parameters.m file. For the model simulation, initial concentrations of all substances, as well as growth and division settings should be defined. The user also sets the number of calculation steps in order to define the time of investigation. If automatic parameter fitting is going to be omitted, the user can optionally set the parameters for the ODE system in the model_parameters.m file. All default example model parameters are consistent with the default ODE system and calculation procedure

**Fig. 2.**
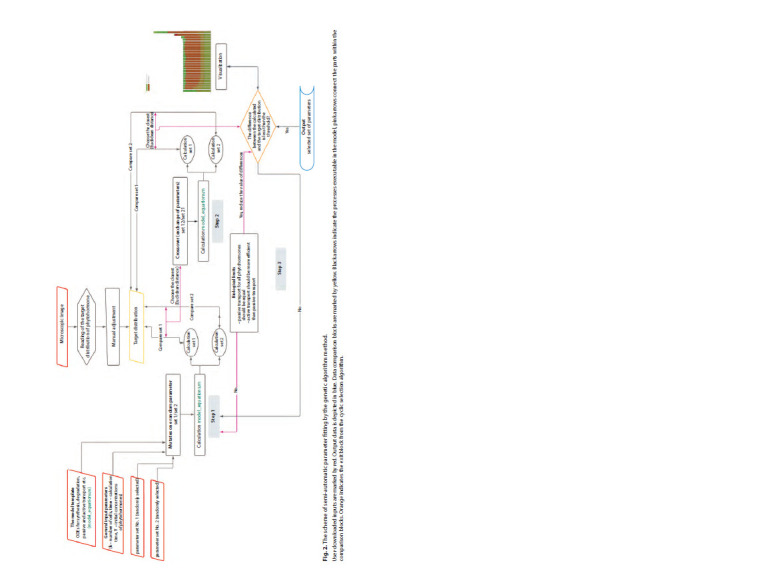
The scheme of semi-automatic parameter fitting by the genetic algorithm method User-downloaded inputs are marked by red. Output data is depicted in blue. Data comparison blocks are marked by yellow. Black arrows indicate the processes executable in the model, pink arrows connect the parts within the comparison blocks. Orange indicates the exit block from the cyclic selection algorithm.

The parameter fitting. First, the parameters_fitting.m script quantifies the distribution of the substance concentration along the selected axis from the microscopic image. These data will be used as target distribution, which the algorithm should reproduce as precisely as possible according to the model equations and configuration. At this stage, the concentration distribution can be manually corrected if it is distorted in the microscopic image. Next, the genetic algorithm is used to find a set of model parameters, which allow reproducing the input experimental data on the distribution of the substance concentration the most accurately (Fig. 2) (Dubitzky et al., 2013).

Initially, the parameters_fitting.m script generates individuals: the sets of model parameters assigned to random values. Each individual is characterized with the fitness function value that scores the similarity of the modeled distribution of the substance concentration to the experimental data. The rootmean- square deviation (RMSD) metric is used as a fitness function. A lower fitness value corresponds to a better quality of the solution. The genetic algorithm is implemented in the following three steps.

Step 1 is “mutation”, which changes a randomly selected parameter in each parameter set by the value of λ (which is also randomly selected in the interval from 0 to 1). For each individual, we calculate the model with a new parameter set. “Mutation” is fixed if it brings the solution closer to the target distribution. Step 2 is “crossover”, the exchange of the parameter values between two individuals. In the first new set of parameters, a few (the number is defined randomly at each step of the algorithm) are picked from individual 1, and the rest are taken from individual 2. The second new set of parameters incorporates the values for the corresponding few parameters from individual 2, and the values for the rest of the parameters are taken from individual 1. The model is calculated with two new sets of parameters after the “crossover”, and the recombination event is fixed if it brings the solution closer to the target. Step 3 supports biologically reasonable limitations on parameter values, which the user can set up manually in the “Biological limits” block of the parameters_fitting.m script (see Fig. 2). The restrictions may apply, for example, to the parity of the passive transport of different substances, the parity of active and passive transport of the same substance, the parity of the substance inflow and synthesis, etc. Taking into account reasonable biological restrictions, the algorithm “rewards” the realistic parameter values during selection, which both favors identification of the local optimum corresponding to the real processes, and speeds up the algorithm.

The fitting ends when the difference between the substance distribution calculated with the adjusted parameters and target substance distribution from the microscopic image becomes less than the threshold. The selected parameters set (the “Par” variable) is saved in a file. After executing the parameters_fitting.m script, it is recommended to inspect the selected parameters, since not all biological limitations could be taken into account during the selection. The user can view the “Par” variable and, if there are obvious inconsistencies in parameter matching, restart the parameter fitting.

**Fig. 3.**
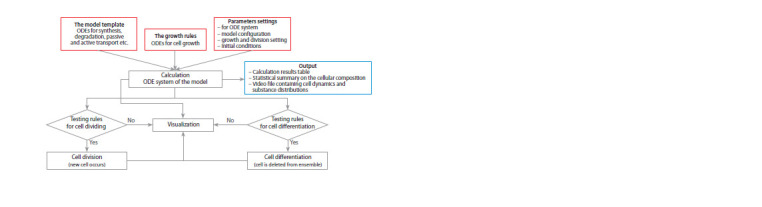
The framework for calculation of the ODE system on a dynamic cell ensemble. Input data are marked by red. Output data are depicted in blue.

Calculation of the mathematical model. When the ODE system and cell growth rules are defined, the user can load the mandatory parameters of the model with the model_parameters() function, including the initial number of cells, the initial concentrations of substances, the initial cell sizes, the maximum number of cells to be monitored, cell division parameters and cell growth settings according to the function described in the grow_eq.m file. Then the user uploads the set of parameters for the model ODE system, which are either obtained during the parameter fitting procedure or defined manually in the model_parameters.m script. After that, the model can be calculated (Fig. 3). We proceed under the assumption that cell dynamic events such as division or differentiation are discrete processes. Therefore, the calculation of ODEs is periodically interrupted to check if the conditions for cell division and differentiation specified in the tool_1d_model.m and model_parameters.m files are met. Optionally, the user decides which substances will regulate the ability to divide and the probability of cell division. All calculation results obtained during the simulation of the model are recorded in a video file, which represents the redistribution of the substance concentrations on a one-dimensional dynamic cellular ensemble.

Images used in the study. To model root apical meristem, we used publicly available images for 9-day-old Arabidopsis thaliana seedlings expressing DR5::GFP auxin sensor (Ottenschläger et al., 2003) or TCSn::GFP cytokinin sensor (Zürcher et al., 2013), which were obtained using a confocal fluorescence microscope (FV-1200, Olympus) (Sakamoto et al., 2019). To model shoot apical meristem, we took publicly available images for 7-day-old A. thaliana seedlings expressing TCSn::GFP cytokinin sensor obtained by a confocal microscope (Leica) (Zürcher et al., 2016). As a visualization of auxin distribution in the shoot apical meristem, we used images of auxin immunolocalization in the inflorescences of 22-day-old A. thaliana seedlings taken by a confocal microscope (LSM, FluoView1000, Olympus) (Banasiak et al., 2019).

## Results

A one-dimensional model of Arabidopsis thaliana root apical meristem built with DyCeModel

**Fig. 4.**
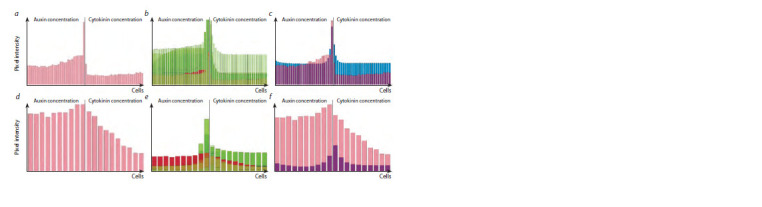
DyCeModel solutions on auxin and cytokinin distribution within the root (a–c) and shoot (d–f ) apical meristems along the central axis. a, d, Obtained signal intensity of hormone distributions along the allocated area; b, e, visualization of the parameter fitting process; c, f, the result of automatic parameter fitting. Pink or burgundy indicates the signal intensity distribution obtained from the experimental data. The distributions of phytohormones during each step of parameter selection are indicated in green. Blue marks the distribution of the substances when calculating the model with the automatically selected set of parameters.

To demonstrate the performance of DyCeModel, we used it to create a 1D model of A. thaliana root apical meristem. Plant hormones auxin and cytokinin play major roles in regulation of maintenance of its structure (Yamoune et al., 2021). We built an ODE system based on mathematical models of auxin and cytokinin distribution published earlier (Mironova et al., 2010; Lavrekha et al., 2014). To set the parameters for the model ODE system, we used automatic parameter fitting. To define the target distribution of the hormone concentrations in the root tip, we used publicly available microscopic images described in the “Materials and methods” section. We used the following parameter value limitations during parameter fitting: approximately equal diffusion parameter values for auxin and cytokinin, prevalence of active auxin transport over the passive transport, prevalence of auxin flow into the meristem over its biosynthesis, which is typical for the root apical meristem (Overvoorde et al., 2010). Figure 4, a–c demonstrates auxin and cytokinin distributions in the root apical meristem generated using the DyCeModel tool. The equation describing the dependence of cell growth on auxin was built on principles similar to the Hartmann model (Hartmann et al., 2021), where the growth rate is directly proportional to auxin concentration in the cell and inversely proportional to the cell size. Cell division can occur if the cell attains minimum size required for division and possesses a certain ratio of auxin and cytokinin concentrations. The probability of cell division is 0.1, the values were obtained by analyzing images and 24-hour videos with marked division events in the meristem of A. thaliana (Marhava et al., 2019). Cell differentiation occurs if the cell size approaches the “maximum cell size” parameter value.

Then we built a functional model of the root apical meristem and obtained a stationary solution. Analysis of the steady-state solution of the root apical meristem model showed that it is consistent with experimental data (García-Gómez et al., 2017; Hu et al., 2021). The distribution of auxin had the shape of an inverted dome and reached a maximum in cells representing the quiescent center. The concentration of cytokinin decreased nonlinearly towards the stem cell niche and reached a minimum in the initial cells, which corresponds to experimental data. In the constructed model obtained with DyCeModel, the correct location and size of the zone of high proliferative activity were specified and remained stable for a long period of calculation, corresponding to those in the root meristem in vivo. Similar zones of proliferative activity were also formed in two other models of the root apical meristem (Mironova et al., 2010; Lavrekha et al., 2014).

DyCeModel enables modeling distinct types of plant meristems based on the same ODEs

We speculated that recruiting specific input characteristics for distinct meristems could enable the modeling of distinct types of plant meristems with DyCeModel based on the same ODEs. Therefore, we applied DyCeModel to build a model of A. thaliana shoot apical meristem using the same mathematical model equations and rules as for root apical meristem. The images used for automatic parameter fitting are described in the “Materials and methods” section. We used the following parameter value limitations during parameter fitting: approximately equal diffusion parameter values for auxin and cytokinin and a low level of auxin synthesis. In the model of the shoot apical meristem, we obtained a hormone distribution profile that qualitatively corresponds to experimental data (Heisler, Jönsson, 2006). Auxin and cytokinin concentrations decreased nonlinearly with distance from the stem cells. One-dimensional simulations of the shoot apical meristem of A. thaliana established a dynamic balance between dividing and differentiated cells. In this way, zones of proliferative activity were identified, and the number of cells within this zone was maintained at a certain level throughout the entire model calculation. At the same time, the identified parameters of passive transport, degradation, and growth were the same for the model of shoot meristem and root meristem, and the parameters determining cell division remained similar.

## Conclusion

The DyCeModel tool constructs mathematical models of hormone distribution based on the processes of their synthesis, degradation, diffusion and active transport in a dynamically developing cellular ensemble. Such models are necessary to consider the influence of hormone distribution on cell growth and division. The developed DyCeModel tool is quite flexible, it provides embedding, addition, mixing of already existing mathematical models. Adding each model to the scripts switches on machine selection of unknown parameters, which speeds up the work with the model and makes it more stable. In addition, DyCeModel makes a statistical summary on the cellular composition that can be used for predictions about the influence of hormones on proliferative cell activity

Using DyCeModel, we built a functional model of the root apical meristem, which was consistent with the experimental data. Next, we applied DyCeModel to build a model of the shoot apical meristem using the same mathematical model equations as for the root apical meristem model and demonstrated that the parameters of passive transport, degradation, growth, even the parameters determining cell division remain similar between root and shoot models. The resulting onedimensional models can be further used as a framework to study the role of hormone-controlled gene networks in cell dynamics in two types of meristem.

## Conflict of interest

The authors declare no conflict of interest.
